# Achievements and Challenges of the Healthcare System in China

**DOI:** 10.7759/cureus.39030

**Published:** 2023-05-15

**Authors:** Cui Chen, Mao Liu

**Affiliations:** 1 School of Clinical Medicine, Affiliated Hospital of North Sichuan Medical College, Nanchong, CHN

**Keywords:** basic medical insurance, public health, health policy, healthcare insurance, global healthcare systems

## Abstract

China’s healthcare system has made great achievements in the management of medical services and public health challenges for the Chinese people. However, the issue of an aging population in Chinese society is becoming more and more salient. The gap between demand and supply of healthcare is expanding. China’s healthcare system is facing unprecedented challenges. These problems include an insufficient medical insurance fund, nonuniform insurance reimbursement policies, a poor integrity system, and a lack of supervision in the management of the medical insurance fund. To address these challenges, some practical solutions are worth considering. A national medical insurance supervision platform should be strengthened. Besides, blacklists for illegal medical institutions and individuals engaged in malicious medical disturbances should be created. The country should also introduce policies to narrow the differences in regional medical insurance policies and balance the reimbursement levels of residents in different regions. Big data and artificial intelligence can be used to monitor the entire process of medical insurance fund utilization. The government needs to establish relevant laws and regulations to optimize the medical insurance system and ensure the safe and effective operation of the medical insurance fund.

## Editorial

A good healthcare system is an indispensable part of the happiness of residents in a country [1]. Nowadays, the existing health systems all over the world are different due to the different combinations of components. China’s healthcare system has gained internationally recognized achievements in the management of medical services and public health challenges for the Chinese people. However, as the aging of the population becomes increasingly serious, more and more elderly people have higher demands for public medical treatment. China’s healthcare system is facing unprecedented challenges [2]. In this editorial, we propose some problems and solutions for the healthcare system in China.

Achievements of China’s healthcare system

The Chinese government has established a sound healthcare system. China is the largest developing country in the world, and it’s very difficult for the government to establish a large and effective healthcare system [2]. With the continuous efforts of successive governments, a total of 1,030,935 healthcare institutions will have been established on the Chinese mainland by the end of 2021. There are 36,570 hospitals and 977,790 primary-level clinics. Hospitals in China are organized according to a three-tier system that recognizes a hospital’s ability to provide medical care, provide medical education, and conduct medical research. Based on this, hospitals are designated as primary, secondary, or tertiary institutions. There are 3,275 tertiary hospitals, which are usually affiliated hospitals of universities or large medical centers in provinces or big cities. There are 10,848 secondary hospitals, which are medium-sized hospitals, in the districts and counties. There are 12,649 primary hospitals and 9,798 unclassified hospitals, which are usually private hospitals. Except for the different-level hospitals above, there are also 36,160 community health service centers in urban areas and 34,943 township health centers in rural areas [3]. This kind of health center is usually a clinic for the basic medical needs of the community and rural residents. Every village has a clinic, and every town or township has a health center served by licensed doctors.

In 2021, the number of beds in medical institutions across the country reached 9.4 million, including 7.4 million in hospitals, 1.7 million in township-level health centers, and 0.3 million in public health institutions. Besides, there are a total of 67,153 ICU beds nationwide [3]. As a result, the country has built an integrated healthcare system of high quality and efficiency and improved the availability and accessibility of medical resources.

The Chinese government has also established a healthcare insurance system that covers almost 96% of the population and benefits more than 1.36 billion people. This system includes basic medical insurance conducted by the government and commercial health insurance provided by various companies. Basic medical insurance is a great-covered social security course and collects the funds from enterprises and individuals. It consists of different insurances for working urban residents, non-working urban residents, and the rural population. Working urban residents pay the most insurance funds and have the highest reimbursement ratio, followed by non-working residents and the rural population [4]. As for the poor and vulnerable individuals, there is a special fund called urban-rural medical aid for serious illnesses, which covers those suffering from economic difficulties. As for the rich, they can also choose to purchase commercial health insurance individually to receive better medical service. As a result, more than 95% of China’s population is covered by the public insurance system [3,4].

China’s healthcare budget is gradually increasing. The ratio of health expenditures to GDP in 2021 was 6.5%. It was only 4.3% to 5.2% during the period from 2000 to 2010. The total national health expenditure in 2021 will be 1184.9 billion dollars. The government's health expenditure is 324.7 billion dollars, accounting for 27.4%. Social health expenditures are 531.7 billion dollars, accounting for 44.9%. Personal health expenditures reached 328.4 billion dollars, accounting for 27.7%. The health expenditure per capita is 838.3 dollars [3].

Based on policies and budget support from the government, China’s healthcare insurance system has effectively reduced the personal financial burden incurred by medical expenses. For example, China’s healthcare insurance system offered free COVID-19 vaccines to all inhabitants. Up to December 2021, more than 1,203 million people had received the COVID-19 vaccination. This is the largest immunization program in human history [3].

Challenges faced by China’s healthcare system

Medical insurance reimbursement policies vary in different provinces or cities according to the level of economic development. The payments for medical insurance from individuals and enterprises are extremely low and have lagged far behind economic growth. What’s worse, due to the lack of long-term vision among some employees, they increase their monthly income by not paying medical insurance fees. Hence, the medical insurance fund reserves are generally insufficient [5]. Besides, the healthcare insurance system implements unified planning at the county level in most parts of China. The policy is not uniform and lacks a swap mechanism, resulting in unfairness and conflicts between people from different regions.

The distribution of medical resources in China is uneven. High-quality medical resources are mainly concentrated in large and medium-sized cities. North China has the most uneven distribution of medical resources. Most of the top 100 hospitals in northern China are concentrated in Beijing. The top hospitals in the western region are concentrated in Xi'an, Chengdu, and Chongqing. Although the economy in South China is relatively developed, medical resources are mainly concentrated in Changsha, Wuhan, and Guangzhou. The number of hospitals in Hainan Province is relatively small [6].

The construction of China’s integrity system is not good enough. Whether for insured individuals or healthcare practitioners who provide clinical activities and medical insurance services, China lacks the necessary integrity supervision and disciplinary measures for dishonesty. In the past two decades, nonstandard medical events and the extraction of medical insurance funds have occurred frequently. For example, some insured individuals apply for medical insurance benefits with false materials. Some other patients intentionally delay discharge and undergo repeated examinations and treatments. Besides, by cooperating with patients, a few hospitals assist patients in applying for fake hospitalization procedures to defraud medical insurance funds [7]. Moreover, some medical institutions or doctors fabricate false medical records, repeatedly charge fees, and falsify medical services to reap profits from medical insurance funds [8].

The capacity of China’s medical insurance information system is also insufficient. At present, about 90% of the medical insurance-coordinated areas in China have established corresponding medical insurance information management platforms. The remaining 10% of areas have not been established yet [9]. Additionally, each province has its own independent medical insurance information code for every medical operation or examination. As a result, there is no unified standard nationwide, and the sharing of medical insurance information has not been achieved. Sometimes, the same project can be reimbursed in one province but not in another. Worse still, most medical insurance employees do not have a medical background. They are usually unclear about the disease and the relative diagnostic and treatment items. The result is that some insured individuals who should be entitled to benefits have not been approved, which is very common in the medical reimbursement process. This can easily lead to inappropriate behavior and increase the risk of incorrect payments [10].

What can we do？

A national medical insurance supervision platform should be strengthened. According to the plans of the Chinese government, a national medical insurance information platform was supposed to be launched by 2020. Due to the COVID-19 pandemic and various other reasons, this system is not yet very smooth and widely applied. Moreover, the supervision of medical behavior and the illegal expenditure of medical insurance funds is not yet very precise and efficient. Therefore, in order to address the issues raised above, we suggest that some improvement measures be taken into consideration as soon as possible.

Firstly, medical insurance rounds and medical insurance fee checks should be conducted regularly to eliminate any violations of medical insurance management regulations that may occur during daily medical treatment and diagnosis.

Secondly, a blacklist system for medical insurance integrity should be created. We are curious why the Chinese government has always been unwilling to establish blacklists for illegal medical institutions and individuals engaged in malicious medical disturbances [11]. In our opinion, those medical institutions or individuals who engage in illegal activities should be severely sanctioned.

Besides, it is necessary to improve the medical knowledge of medical insurance staff. When recruiting new employees, a medical background should be taken into consideration. Medical insurance employees should receive regular training on basic medical knowledge about diagnostic and treatment items.

Furthermore, in the operation process of medical insurance funds, it is necessary to continuously improve the information supervision mechanism and make reasonable use of modern information technology, big data, and artificial intelligence to monitor the entire process of medical insurance fund utilization, from manual mode review to system automation overall coverage.

Last but not least, the country should introduce policies to narrow the differences in regional medical insurance policies and balance the reimbursement levels of residents in different provinces or regions. What is more important is that further optimization of government subsidies and individual payment structures is needed to enhance the sustainability of medical security funds. This is the main measure to solve the problem of insufficient medical insurance funds.

In summary, the Chinese government has built a sound and efficient healthcare system, which has improved the accessibility of medical resources and reduced the medical burden of people to a certain extent. On the other hand, there are still problems with the risk control of China’s medical insurance system and the insufficiency of medical insurance funds. Therefore, it is necessary to establish relevant laws and regulations to optimize the medical insurance system and ensure the safe and effective operation of the medical insurance fund.
